# Social impacts and post-disaster management in disaster-prone areas of East Java, Indonesia

**DOI:** 10.4102/jamba.v16i1.1747

**Published:** 2024-11-20

**Authors:** Karnaji Karnaji, Emy Susanti, Septi Ariadi, Muhammad Saud

**Affiliations:** 1 Department of Sociology, Faculty of Social and Political Sciences, Universitas Airlangga, Surabaya, Indonesia

**Keywords:** disaster, disaster prone, disaster impact, disaster victim handling, Indonesia

## Abstract

The present study seeks to identify disaster-prone areas and socio-economic impacts to investigate the mechanism of the post-disaster management in East Java, Indonesia. The data was collected from three disaster-prone areas, i.e. Lumajang, Malang, and Bojonegoro regencies in East Java. This study applied the mixed method approach; hence, quantitative (survey) and qualitative (interviews) data have been gathered. The results show that disasters have several implications for human life, such as health, economic life, and socio-cultural and psychological conditions, which have several impacts on communities, such as human settlements, health, economic life, and socio-cultural and psychological conditions. Therefore, efforts are required to manage disaster victims and provide them with social protections related to mental, economic, and social activities and health services.

**Contribution:**

In disaster-affected areas, to restore the community’s economic condition, local governments provide multiple kinds of assistance, including business capital (grants), low interest rates, production equipment, community empowerment activities, job opportunities and business partnerships with the local market. Furthermore, such strategies for disaster management should be implemented in an integrated manner, and the inclusion of local community members for prevention, mitigation, preparedness, and emergency response to recovery phases are also highly recommended.

## Introduction

Disasters in Indonesia can be attributed to several factors, both natural and human-made (Gan et al. 2021; Pribadi et al. 2021). The natural factors include the country’s location on the Pacific Ring of Fire, an area characterised by high volcanic and seismic activity. Indonesia sits at the meeting point of several tectonic plates, making it highly susceptible to earthquakes, tsunamis and volcanic eruptions (Rindrasih et al. 2019). Additionally, the country is prone to extreme weather events such as floods, landslides and forest fires, which can be exacerbated by climate change. Human-made factors contributing to disasters in Indonesia (Harrowell & Özerdem 2019) include poor infrastructure, inadequate building planning and urbanisation in high-risk areas (Regita & Hadi 2022). Many buildings in Indonesia are not constructed in accordance with regulations designed to mitigate the impact of natural hazards, rendering them more vulnerable to collapse during earthquakes or floods. Furthermore, the rapid growth of urban areas has led to the development of housing in high-risk locations, such as alongside riverbanks, which increases the risk of landslides and flooding (Gunawan et al. 2015). Consequently, the capital of Indonesia, Jakarta, is significantly affected by unplanned urbanisation. Indonesia has a large population and is one of the world’s most densely populated countries. As a result, many communities in Indonesia face the effects of disasters, leading to higher casualties and greater damage to infrastructure.

To mitigate the risk of disasters, Indonesia has implemented several measures such as building better infrastructure, improving building planning, and developing early warning systems for earthquakes and tsunamis. However, there is still more work to be accomplished to ensure the population’s safety and to prepare some more applied strategies to minimise the impact of future disasters. One of the factors behind the disasters is the impact of global warming, which is because of human behaviour (in Bahasa Indonesia; *Nggolek, enak-e, dhewe*). It is not only affected in Indonesia but also globally. Some strategies to reduce the chances of disasters caused by human actions include large residential communities, big shopping malls and industries, which are typical in Indonesia. Therefore, to reduce the effects of global warming, multiple strategies can be employed, such as increasing the plantation of mangrove areas along the coast and expanding forests in upstream watersheds. This research aims to identify strategies and mechanisms for disaster management in three regions of Java, Indonesia. The emergency anticipation programme related to disaster management strategies did not adequately analyse the conditions of disaster-prone areas. Additionally, the formulation of this emergency anticipation programme was insufficient. As a result, government infrastructure lacks sufficient capacity to handle disasters. Areas prone to natural and industrial disasters typically do not have the resources to raise awareness about managing disaster emergencies (Ahmad 2002; Kodra & Syaukani 2004; Simanungkalit 2022).

While conceptualising the term ‘disaster,’ it refers to an event or series of events caused by nature, humans or both that result in human suffering, loss of property, environmental damage, damage to infrastructure and public facilities, as well as disruption of the life and livelihoods of the community (Purworini, Hartuti & Purnamasari 2021; Shaluf & Ahmadun 2006; Simanungkalit 2022). In various regions, the impact of disasters can be summarised in five points. Firstly, large-scale natural hazards cause significant damage to houses. For example, during floods, landslides or hurricanes, homes may be submerged in water or collapse because of strong winds. Secondly, these disasters can devastate tens of hectares of productive land, rendering farmers who are unable to do the plantations. Thirdly, disasters often damage many public facilities, including schools, bridges, roads and other essential infrastructure. Fourthly, community members may lose their livestock or production assets, leading to health problems and moralities. In many cities and districts, disasters not only force some residents to evacuate and seek shelter, but extreme events often also result in trauma, serious injuries and even death (Kodra & Syaukani 2004; Kusumasari & Alam 2012; Lan Huong et al. 2022; Simanungkalit 2022; Widianarko & Pandingan 2002). Numerous factors contribute to flooding in various areas; however, the various flood disasters in recent years are directly or indirectly linked to global warming and human actions that neglect long-term environmental sustainability (O’Brien et al. 2010; Schneider 2011).

The present study is being conducted in East Java and is divided into four subareas. The first is the middle area, which is the most fertile area from Ngawi to Banyuwangi. The second is the northern area, which is moderately fertile, spanning from Bojonegoro and Tuban to Madura Island. The third area is the southern region, from Malang to Pacitan. Lastly, the fourth area encompasses the infertile regions, including Gresik, Probolinggo, Sampang and the Archipelago of Sumenep. East Java is not immune to disasters; it is usually affected by various disasters, such as floods and hurricanes (Haerani, Muslim & Muslim 2017), which tend to occur relatively evenly across different regions, particularly during the rainy season. Floods can result from excessive rainfall, overflowing rivers, fragile levees and water flowing from hillsides, leading to landslides and flash floods. These issues can also stem from human activities and miscalculations in urban planning (Helmi et al. 2019). In coastal areas, flooding is threatened by tsunamis and high tides, while disasters in coastal village communities are influenced by strong winds and sea waves during the rainy season (BPS 2015; Sagala et al. 2021; Sosilawati et al. 2017).

In addition to flooding, the threat of landslides is a significant disaster in the hilly areas of East Java, Indonesia. In Mojokerto, several people have died because of a landslide (Wibowo 2003). The end of the rainy season does not signify the end of disasters; many communities continue to face health issues, particularly malaria and dengue, related to these disasters and floods (BPBD Mojokerto 2014). In Bangkalan, floods have affected 54 ha of agricultural land, resulting in destruction, while 30 ha of ponds have failed to yield a harvest. In Lamongan, flooding has submerged 3000 ha of land. While, in Bondowoso and other flood-affected areas, crop failure and damage to agricultural land are unavoidable.

**FIGURE 1:**
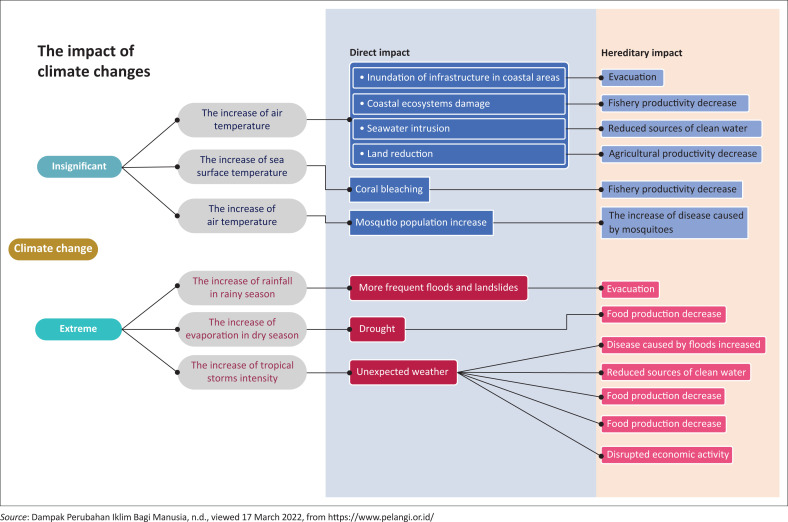
Impacts of climate change.

On the other hand, in the prolonged dry season, farmers fail to irrigate their fields (BNPB 2022; Suryanto & Rahman 2019). Furthermore, disasters have severe impacts, including health problems, forced evacuations, trauma, serious injuries and even death (Figure 1). If these issues are not addressed immediately, the suffering experienced by victims and the burden on the community will only increase (Kusumasari & Alam 2012; Purworini et al. 2021; Shaluf & Ahmadun 2006; Soegijoko & Kusbiantoro 1997).

The circumstances and impacts of disasters should be addressed with strategies to combat such situations. Therefore, disaster management is conducted in a planned, organised and continuous manner, incorporating new parameters for disaster observation. This approach fosters risk assessment, prevention, mitigation, preparedness, early warning, emergency response, restoration and reconstruction within the context of safety and development. The aim is to reduce the likelihood of victims and damage, eliminate suffering and alleviate the difficulties faced by those affected. It also seeks to facilitate return migration to disaster areas and ensure the provision of livable conditions, safe drinking water, electricity and other communication channels, while restoring the economic and social life of the affected region (Nyahunda, Tirivangasi & Mabila 2022; Xue et al. 2022). Additionally, the aspects of disaster management include addressing the physical, mental and social needs of natural hazard victims as effectively as possible, in accordance with local conditions. This involves increasing the capacity, motivation and engagement of victims in restoration, rehabilitation and reconstruction activities, as well as addressing the psycho-social issues they face and enhancing their social roles. In such cases, it is crucial to prevent and mitigate the various losses experienced by victims during impending disasters (Ahmad 2002; Inoguchi et al. 2003).

The disaster management cycle consists of phases based on specific situations or conditions, including preparedness, emergency response, prevention and post-disaster mitigation. The disaster management strategies developed are generally adaptable and vary according to field circumstances. These phases are:

- Prevention, where efforts should be focussed on reducing the likelihood of damage and minimising the number of victims;
- Emergency response, which involves search and rescue operations as well as the provision of emergency assistance (such as food, clothing, shelter, medicines and more);
- Rehabilitation, which includes both physical and non-physical assistance aimed at empowering and restoring the lives of disaster victims; and
- Reconstruction, which involves rebuilding infrastructure and public facilities to help disaster victims return to normalcy (UU No. 24 Tahun 2007 Tentang Penanggulangan Bencana).

## Research methods and design

This research was conducted in multiple stages, focussing on disaster-prone regions of cities and districts in East Java Province. The present study employed a mixed-method design, collecting data through both qualitative and quantitative research approaches. Data from previous studies and relevant literature on disasters were also included in the data collection and analysis process. This study was based on surveys (quantitative) and interviews (qualitative), with primary data obtained through field activities in three East Java regions: Malang, Lumajang and Bojonegoro. These research sites were chosen because of the diversity of the population and the frequency of disasters in the area. Data were gathered through interviews and surveys, with more than 175 participants approached using a simple random sampling technique; however, only 150 were included in the data analysis and processing for this research. Participants were approached by visiting the homes of individuals who had been victims of disasters. For data collection, an interview guide and a survey questionnaire were prepared to compile the results. The data were categorised through editing, entry, processing and analysis using SPSS v.26 software. The results were analysed and interpreted, leading to several important conclusions related to efforts in assisting victims of natural hazards.

## Results and discussion

### Profile of disaster-prone areas in East Java

East Java has a tropical climate that recognises two seasonal changes: the rainy season (October–April) and the dry season (May–September). The average temperature is at a minimum of 15.2 °C and a maximum of 34.2 °. Further, according to the Schmidt and Ferguson classification system, most areas (52%) have a type D climate. The humidity ranges from 40% to 97%. The average rainfall is 1500 mm/year to 2700 mm/year. Precipitation per year in East Java has three characteristics, namely: (1) less than 1750 mm/year covering an area of about 35.5%; (2) between 1750 mm and 2000 mm/year covers an area of about 44% and (3) above 2000 mm/year covers an area of about 20.46%. Meanwhile, the average wind speed moves between 6 and 45 knots. Regional topography in East Java is divided into <500 masl by 86% (BPS 2015).

In terms of topography, East Java Province is home to 48 mountains, including five active volcanoes: Mount Semeru, Mount Kelud, Mount Bromo, Mount Raung and Mount Lamongan. Hydrologically, East Java is divided into four River Basin Units (SWS):

- Madura SWS, with a potential water storage capacity of about 10 million m^3^;
- Pekalen Sampean SWS, with a potential water storage capacity of around 21.85 million m^3^;
- Brantas SWS, with a potential water storage capacity of approximately 505.70 million m^3^; and
- Bengawan Solo SWS, with a potential water storage capacity of around 142.45 million m^3^.

The slope classification in East Java is divided as follows: areas with slopes greater than 60% are classified as hilly and mountainous; areas with slopes between 30% – 60% are classified as medium highlands and areas with slopes less than 20% are classified as lowlands (Blomquist, Dinar & Kemper 2005).

Natural conditions such as climate, wind speed, topography, volcanic activity and the hydrology of large rivers, along with marine life and potential hazards, can become income resources for the population if managed appropriately. For example:

- One of the essential factors affecting soil fertility is the number of active volcanoes and the flow of large rivers. Volcanoes and river flows facilitate the dispersal of nutrients contained in materials resulting from volcanic eruptions;
- The Centre for River Basin Water Resources Management (BPSAWS) is responsible for managing river water resources (see Table 1).

**TABLE 1:** Centre for River Water Resources Management (BPSAWS) in East Java.

Centre location	Area coverage
Madura	All districts in Madura Island
Buntung Paketingan	Mojokerto, Surabaya and Sidoarjo
Gembong Pakelan	Pasuruan and Probolinggo
Mayang Bondoyudo	Lumajang and Jember
Sampean Baru	Situbondo, Bondowoso and Banyuwangi
Bengawan Solo	Bojonegoro, Tuban, Lamongan and Gresik
Bango Gedangan	Malang, Blitar, Tulungagung and Trenggalek
Selodono	Kediri, Ngajuk and Jombang
Madiun	Madiun, Ponorogo, Magetan and Ngawi

*Source:* Dpunair, 2019, *Balai besar wilayah sungai berantas*, viewed 17 March 2022, from https://sda.pu.go.id/profil/informasi_balai/balai_besar_wilayah_sungai_berantas.

Its functions include meeting the water needs of the agricultural sector and controlling floods (Rodgers & Zaafrano 2002; Subijanto & Valiant 2009).

Out of the nine resource management centres, only two – Brantas and Bengawan Solo – are managed in an integrated manner. In the case of Bengawan Solo, management occurs across the provinces of East Java and Central Java. Administratively, of the 38 cities and regencies in East Java Province, nearly all have been affected by disasters. Natural hazards are unavoidable and cannot be predicted accurately (Purworini, Purnamasari & Hartuti 2019). However, regardless of a disaster’s severity, its impact can be mitigated, and the associated risks can be reduced if appropriate anticipatory measures are taken. Residents in disaster-prone areas must ensure they are adequately prepared.

In East Java, floods caused by overflowing water from the Bengawan Solo River have occurred at least twice in the last 3 months. This river, the longest on Java Island, stretches approximately 600 km. It is divided into three main sub-watersheds: the Upper Solo Bengawan Sub-watershed, with an area of 6072 km^2^; the Kali Madiun Watershed, covering 3755 km^2^ and the Bengawan Solo Downstream Sub-watershed, which spans 6273 km^2^. Water from the Upper Solo Sub-watershed and the Kali Madiun Sub-watershed converges in Ngawi before flowing downstream.

The development funds to maintain the Solo River are often insufficient. Levees along the Bengawan Solo River, which should be maintained for their durability and strength, are frequently under-implemented or completed in limited numbers. Additionally, certain sections of the levee become brittle because of ongoing water erosion. During periods of heavy rainfall, these fragile levees can be torn apart by flooding. At the end of 2020, 22 regions in East Java were identified as prone to hydrometeorological disasters during the peak of the rainy season, including hurricane floods, extreme weather and landslides. According to the Regional Disaster Management Agency (BPBD) of East Java, these 22 regions are at risk of flooding from overflowing water from the Bengawan Solo River and other areas (BPBD Jawa Timur 2022).

The occurrence of hydrometeorological disasters poses a threat to various mountainous and hilly regions, with potential impacts on many other areas as well. At least 22 locations in East Java are prone to such disasters, including flash floods, landslides and typhoons, especially during the rainy season. Therefore, it is essential to remain vigilant and prepared for the threats associated with hydrometeorological disasters, particularly those stemming from the La Niña phenomenon, which can lead to floods, landslides and tornadoes (BNPB 2022).

Based on the data, it can be seen that East Java Province is one of the provinces that are geographically vulnerable to disasters. Therefore, disaster management must be conducted through synergistic and intense collaboration between local and provincial entities. Vigilance can be enhanced by focussing on the Meteorology, Climatology and Geophysics Agency (BMKG), which regularly issues early disaster warnings. These alerts are crucial for raising community awareness and increasing preparedness, especially for those living in disaster-prone areas. Additionally, district and city governments need to implement various disaster management mechanisms to better anticipate and address challenges, minimising the prolonged impacts on communities (Sibanda, Mukwada & Hansen 2022).

### Impact of natural hazards experienced by respondents

In this study, based on observations made during field trips, flooding is identified as a natural hazard that frequently occurs in various areas of East Java. Such flood disasters typically arise during the rainy season, characterised by high rainfall intensity, which leads to an increase in river water discharge. Floods impacting residential areas and agricultural land have created significant challenges for the communities living near the river.

In Table 2, similar incidents were also experienced by victims of other natural hazards such as earthquakes, volcanic eruptions, volcanic ash, rain, landslides, hurricanes and more (Table 2). As a result of these various types of natural hazards, the community has suffered losses on both small and large scales. The extent of damage caused by these natural hazards varied: 38% reported severe damage, 11.3% reported moderate damage and 27.3% reported minor damage.

**TABLE 2:** Kind of disaster experienced.

No.	Impacts of disasters and types	%
**A**	**Type of disasters**	
1	Landslide	30.00
2	Flood	64.73
3	Hurricane	22.70
4	Earthquake	58.00
5	Cold lava	28.70
6	Erupting volcano	26.70
**B**	**Damage and losses**	
1	House damage	58.00
2	Damage to agricultural land/fields	47.30
3	Losing money	10.00
4	Loss of valuables	0.70
5	Loss of livestock	20.70
6	Vehicle damage	18.70
7	Damage to business premises	18.70
8	Household furniture damage	37.30
**C**	**Business impact**	
1	Business interrupted	53.70
2	Decrease in turnover	44.70
3	Land damage	41.30
4	Reduced market share	28.00
5	Broken production equipment	27.30
6	Damaged production materials	36.70
7	Changing jobs	12.70

Disasters have a significant impact on material losses that are difficult to quantify. Residents along the river, in the hills or in the mountains often become victims. The data in Table 2 indicate that the damage caused by natural hazards in the severe category includes house damage (25.3%), damage to business premises (16.0%), damage to household furniture (16.7%), loss of livestock (16.7%) and damage to vehicles (14.7%). Regardless of the absolute amount of material losses experienced by victims of natural hazards, it is challenging to estimate the losses and pain that poor individuals face during these difficult times. When someone loses their house, livestock perishes, agricultural fields flood before harvesting and savings are lost, it is understandable that the community feels worse off. A house, which takes time to build, can be destroyed in just a few hours. Beyond material losses, the impact of flood disasters can stifle the potential for community self-help and resilience.

Furthermore, Table 2 shows that most respondents experienced losses in the nominal range of IDR 500 000 to 5 000 000 (66%). Among the victims, 13.0% suffered heavy damage, with losses exceeding IDR 5 000 000, primarily because of damage to houses and agricultural land. In addition to material effects, respondents reported that some family members experienced health issues (26.7%), were forced to evacuate (24.0%), saw their savings reduced (20.0%) and were unable to meet their daily needs (38.6%). On the days following the disaster, 42.0% of respondents reported experiencing mental stress because of the events.

The most severe loss for farmer groups is the destruction of their agricultural land. In the present study, some respondents reported that their agricultural land was damaged as soon as the rice harvest season began. The purchased rice seeds and fertilisers that had already been planted were ultimately lost because of the floods. For traders, the most significant loss is the unfavourable market conditions resulting from a decrease in purchasing power. Several respondents admitted that their turnover fell drastically because of the reduced market share. Private employees may be somewhat fortunate if their workplace is not flooded; however, their situation changes dramatically if their place of business is also affected. This study found that some respondents were forced to seek new employment because their previous jobs could no longer be relied upon. Additionally, victims of disasters experience psychological effects. The study revealed that some individuals faced traumatic situations, such as shock upon hearing loud noises, persistent anxiety and difficulty sleeping during signs of rain or extreme weather conditions.

The traumatic situation described in Table 3 is reflected in the anxiety and worry felt by individuals living in disaster-prone areas. It was found that 59.4% of the respondents reported experiencing stress and worry. The anxiety expressed by several respondents in this study is understandable, given their past experiences with various natural hazards of considerable intensity. Many have faced difficulties, damage, losses and impacts from these disasters, which extend beyond material losses to include social, psychological, economic and even health and educational challenges, affecting the lives of their families post-disaster. Consequently, it is natural for traumatic feelings and fears of natural hazards to arise and resurface, particularly during the rainy season. For these individuals, this season is seen as more prone to natural hazards compared to the dry season. The rainy season poses the risk of not only floods and landslides but also storms and hurricanes, which can lead to significant losses and threaten the lives of respondents and their families.

**TABLE 3:** Psychological impacts because of natural hazards.

No.	Damage type	%
1	Have you faced trauma?	66.0
2	Feel shocked when they hear the rumble	49.3
3	Afraid of something happening to family members	64.0
4	Anxiety over damaged assets	43.3
5	Worried about losing their jobs	28.0
6	Worried that it will reappear when facing a natural hazard situation	40.0

Moreover, people living along the Bengawan Solo River often experience anxiety and trauma because of the threat of flooding. When a flood occurs, they must flee in a panic, and many of their belongings may be lost. Household items such as furniture, TVs, refrigerators, livestock, motor vehicles, valuables, books, certificates and important documents can be destroyed by the floodwaters. Home furnishings accumulated over many years can be lost in an instant. Similarly, hilly or mountainous areas are vulnerable to landslides, volcanic ash, heavy rain, earthquakes and cold lava floods.

### The government’s handling of disaster victims

Disasters in several areas of East Java Province are events caused by nature, humans or both, resulting in human suffering, loss of land or property, environmental damage, damage to infrastructure or public facilities and disturbances to community livelihoods. Disasters are like nightmares, often unpredictable in their occurrence. They can happen anytime, anywhere and can affect anyone in our global world. In general, disasters have the following impacts:

- damage to standard patterns of life, which is usually severe and always has an impact ranging from low to serious severity;
- harm to humans, manifested in death, loss, injury, disability, misery or other negative consequences;
- disturbances in government systems, communication, transportation and various other public services, especially access to drinking water, electricity and telephones; and
- sudden community needs for shelter, food, clothing, health assistance, clean water and social services.

Therefore, regardless of how small a disaster may be, if the community is unprepared and there is a lack of proper response from the government, the burden of suffering on the victims and the community will be significantly greater (Bako & Ojolowo 2021; Chari & Ngcamu 2019; Kementerian Sosial Republik Indonesia 2012).

There is an understanding of how the respondents cope with disasters in their areas. According to a respondent, after a disaster, they lived in their own homes (62%), stayed in relatives’ houses (10%) and occupied refugee camps (28%). The choice of residence after the disaster closely relates to the severity of the damage to their homes. The duration of evacuation experienced by respondents varied; some evacuated for only one day, while others were displaced for 1 week, 2 weeks, or even up to 1 month, depending on the extent of the damage to their homes and the possibility of safely and comfortably returning.

For example, respondents who experienced a flood disaster that inundated their homes often found the situation so severe that they could no longer stay in their residences. Instead, they temporarily relocated to relatives’ homes or refugee camps until the flood subsided. Similarly, victims of natural hazards such as earthquakes or landslides face comparable challenges. If an earthquake or landslide destroys their house, they typically seek shelter with relatives or in a refugee camp. Meanwhile, some disaster victims who experience minor damage to their homes or property tend to remain at home and seek other resources to cope with the situation.

It is reported in Table 4 that most respondents admitted they generally received relatively good treatment during their stay in the evacuation camp. Eight per cent of children experienced problems continuing their education. However, some respondents reported that their belongings were threatened if they were lost or damaged in the refugee camp (16.6%). Locations and evacuation camps for disaster victims are usually provided by various parties, including village or sub-district officials, district officials, provincial and central government, and private entities. The number of respondents who stated that their needs were adequately met was reasonable because various facilities were available in the evacuation camps, such as sanitation, kitchens, places to rest and sleeping arrangements.

**TABLE 4:** Respondents’ assessment of evacuation camps.

No.	Eligibility level	%
1	Very adequate	7.40
2	Adequate	64.20
3	Quite adequate	7.14
4	Inadequate	4.77
5	Very inadequate	16.68

In a discussion regarding the description of Table 5, previous studies on disasters and families have shown that vulnerable groups are often separated during evacuations (Lan Huong et al. 2022; Lee et al. 2022). Recognising that disasters in various areas of East Java can occur repeatedly, the local government has implemented several activities to mitigate the impact of disasters, aiming to reduce the number of victims and enable the community to take proactive steps. Some of the activities carried out by local governments include providing information (61.3%), conducting periodic follow-up on disasters (59.3%), establishing gathering points in case of an emergency (44%), disseminating information on standard protocols during natural hazards and preparing evacuation locations (40.7%), and offering food assistance in the event of a disaster (48.7%).

**TABLE 5:** Treatment of victims’ evacuation.

No.	Treatment of victims during evacuation	Specially treated (%)	Ordinary (%)	Neglect (%)
1	Woman	24.0	36.7	4.7
2	Children	25.3	35.3	7.3
3	Parent	36.7	24.0	7.3
4	People with disabilities	32.0	29.3	7.3

As shown in Table 6, respondents indicate that the availability of medicines and quality health services is essential, particularly for victims of natural hazards who are injured or require medical treatment. Victims of flood disasters, for example, often experience subsequent health issues such as dysentery, diarrhoea and skin diseases. It is crucial to establish mechanisms for managing disaster victims to mitigate further impacts and prevent loss of life because of natural hazards. This process involves various parties, including local government officials, village and sub-district officials, sub-district and district governments, stakeholders and community members (Arifin et al. 2021; Pratama & Nurmandi 2020). Involving diverse stakeholders is vital to ensure that assistance is provided quickly, fairly and equitably while upholding values of togetherness and eliminating discriminatory behaviour. A relief delivery mechanism based on these principles greatly enhances the effectiveness and efficiency of the aid distribution process.

**TABLE 6:** Condition of facilities provided by the government in the evacuation camp.

No.	Reliefs type	Very adequate	Adequate	Quite adequate	Inadequate
1	Bed	2.7	15.3	57.3	24.7
2	Clothes	6.1	6.3	32.3	55.3
3	Fulfilment of food needs	18.0	25.3	52.3	4.4
4	Availability of clean water	18.0	34.7	39.3	8.0
5	Availability of sanitation	11.3	36.7	45.3	6.7
6	Availability of health services	22.0	37.3	27.7	13.0
7	Availability of medicines	12.0	28.7	37.3	22.0
8	Camp security conditions	32.0	31.3	12.0	24.7
9	Information related to disasters	41.0	30.7	18.0	10.3
10	Availability of transportation	2.0	25.7	30.0	42.3
11	Availability of electricity	2.0	29.3	61.3	7.3

The data presented in Table 7 show that respondents indicated a significant focus on relief efforts for disabled individuals (54.7%) and vulnerable groups (53.3%). It is noteworthy that these relief efforts do not distinguish between social and cultural identities, such as the gender of the victim (75.3%), ethnicity and race (84.6%) and religion (86.6%). This indicates that in disaster rescue, particularly in relief efforts, the principles of justice and non-discrimination are upheld, ensuring that all individuals, regardless of their ethnic, racial or religious backgrounds, receive relatively equal opportunities and treatment.

**TABLE 7:** Mechanisms for the process of providing relief by the government.

No.	Assessment of disaster victims	%
1	Easy and fast	69.3
2	Fair and equitable	62.0
3	Given in an orderly manner	66.7
4	Non-discriminatory	67.3

In addition to offering relief and various facilities to meet the needs of disaster victims, the local government often supports socio-cultural protection by providing opportunities for victims to engage in socio-cultural and economic activities. The government can assist in several areas, including: worship or spiritual activities (42.7%), economic activities (such as selling in markets and shops) (48.3%), social activities (44%), traditional ceremonies (33.3%), educational activities (39.3%), sports activities (15.3%) and health service activities (46.7%).

Protection of various socio-cultural and economic activities, as well as health, is urgently needed for victims of natural hazards so they can resume their socio-economic and cultural lives as before. Some actions taken in response to disaster events are rational and align with cultural values, traditions or customs. Therefore, safeguarding spiritual or worship activities, traditional ceremonies, social gatherings and other socio-cultural aspects is essential. Additionally, protecting productive economic activities is crucial, as the financial sector can provide opportunities for work and income, allowing disaster victims to maintain the continuity of their lives and businesses. Victims of natural hazards need encouragement and motivation to rebuild and sustain their productive economic activities and daily lives after the disaster. Thus, affirmative action is required to support and restore the economic conditions of disaster victim.

## Conclusion

This study concludes that recent disasters in East Java Province have often impacted multiple areas, resulting from both natural and human factors. These disasters lead to human suffering, property loss, environmental damage, infrastructure destruction and disruptions to community livelihoods. However, it is essential to recognise that disaster victims exhibit different patterns and mechanisms of coping with adversity. For instance, traditional disaster management mechanisms rely on the support and assistance of relatives or others who can be approached for help. Consequently, vulnerable communities affected by natural hazards find it challenging to recover independently, as nearly all their energy and productive assets are severely impacted. When their homes are damaged, furniture is destroyed, jobs are lost or cultivated land is devastated, they require assistance from various sources, including the government, relatives, stakeholders and other concerned communities committed to disaster management.

The disaster-prone status indicates that by the end of 2020, only 22 districts in East Java were identified as having this designation during the peak of the rainy season. These areas face a significant risk of flooding from overflowing waters of the Bengawan Solo River, the Brantas River, the Welang River and the Kemuning River. Additionally, the risk of landslides and earthquakes threatens various regions characterised by mountainous and hilly terrain. Other natural hazards include volcanic ash, rain, cold and hot lava floods, and earthquakes. Notably, volcanic ash and earthquakes also occurred because of the eruption of Mount Kelud. In relation to these disaster-prone areas, several respondents reported experiencing landslides, floods, hurricanes, earthquakes, cold lava and volcanic eruptions with varying intensities, ranging from 1–2 occurrences to more than seven. These events have resulted in social, economic and psychological impacts for the victims.

Although the government has made many efforts to assist victims of natural hazards, merely handling emergency rescue programmes is not sufficient. When a disaster occurs, the government and local officials must support victims by facilitating their evacuation, providing shelter and meeting their basic needs. However, there is a pressing need to develop a strong and substantial disaster management system that is more responsive than the paradigms created in the past. The current and future approaches to disaster management must focus on prevention and be supported by existing initiatives. Community participation in disaster preparedness is vital for developing an effective mechanism. Therefore, a community support system, including assistance from the private sector, should be leveraged to reduce risk. Additionally, economic empowerment initiatives for disaster-affected communities are necessary to revive enthusiasm and restore socio-economic conditions by offering various forms of assistance tailored to the needs of disaster victims.
